# Tumor immune microenvironment and immunotherapy efficacy in *BRAF* mutation non-small-cell lung cancer

**DOI:** 10.1038/s41419-022-05510-4

**Published:** 2022-12-21

**Authors:** Hui Li, Yongchang Zhang, Yanjun Xu, Zhiyu Huang, Guoping Cheng, Mingyin Xie, Zichao Zhou, Yangyang Yu, Wenjing Xi, Yun Fan

**Affiliations:** 1 Department of Medical Oncology, the Cancer Hospital of the University of Chinese Academy of Sciences (Zhejiang Cancer Hospital); Institute of Basic Medicine and Cancer (IBMC), Chinese Academy of Sciences, Hangzhou, Zhejiang 310022 China; 2 Department of Medical Oncology, Lung Cancer and Gastrointestinal Unit, Hunan Cancer Hospital/The Affiliated Cancer Hospital of Xiangya School of Medicine, Central South University, Changsha, Hunan 410022 China; 3 Department of Pathology, the Cancer Hospital of the University of Chinese Academy of Sciences (Zhejiang Cancer Hospital); Institute of Basic Medicine and Cancer (IBMC), Chinese Academy of Sciences, Hangzhou, Zhejiang 310022 China; 4 The State Key Laboratory of Translational Medicine and Innovative Drug Development, Jiangsu Simcere Diagnostics Co., Ltd, Nanjing, Jiangsu 210042 China

**Keywords:** Translational research, Predictive markers

## Abstract

Previous small-size studies reported *BRAF*-mutated NSCLC patients have comparable sensitivity to immune checkpoint inhibitors (ICIs). However, how *BRAF* mutation affects the tumor immune microenvironment (TIME) is unknown. We performed Nanostring-panel RNA sequencing to evaluate TIME in 57 *BRAF* mutated and wild-type (WT) NSCLC specimens (cohort A). The efficacy of ICI monotherapy or combined therapies was determined in 417 patients with WT and *BRAF* mutated NSCLC (cohort B). We found that *BRAF*-mutant tumors had similar ratios of CD8+ T cells to Tregs, the balance of cytotoxicity gene expression signatures and immune suppressive features, and similar ICI-response-related biomarkers to WT NSCLC. A similar TIME pattern was observed between the *BRAF* V600E and Non-V600E subgroups of NSCLC. The further retrospective study confirmed that treatment with ICI monotherapy or combined therapies resulted in similar overall survival (OS) (HR: 0.85; 95% CI, 0.56 to 1.30; *p* = 0.47) and progress-free survival (PFS) (HR: 1.02; 95% CI, 0.72 to 1.44; *p* = 0.91) of patients with WT (*n* = 358) and *BRAF* mutant (*n* = 59) NSCLC. Similarly, both patients with *BRAF* V600E or Non-V600E NSCLC had similar responses to immunotherapy. Our findings support that *BRAF* mutation did not modulate TIME in NSCLC and therapeutic responses to ICIs. Patients with NSCLC harboring *BRAF* mutation should not be denied treatment with ICIs.

## Introduction

Lung cancer is the leading cause of cancer-related deaths worldwide, including in China [1]. Molecular-targeted therapies against alterations in several oncogenic driver genes, such as epidermal growth factor receptor (*EGFR*), anaplastic lymphoma kinase (*ALK*), and ROS Proto-Oncogene 1 (*ROS1*, a receptor tyrosine kinase), have significantly improved the prognosis of cancer patients harboring these gene mutations [2, 3]. Furthermore, immune checkpoint inhibitors (ICIs) targeting programmed cell death protein 1 (PD-1), PD-ligand 1 (PD-L1), and cytotoxic T lymphocyte-associated antigen-4 (CTLA-4) has demonstrated to benefit cancer patients in the clinic and emerged as a standard treatment strategy in advanced non-small cell lung cancer (NSCLC) [4]. Unfortunately, clinical data suggest that some NSCLC patients harbored classic gene mutations, such as EGFR mutations and *STK11*/*KRAS* co-mutations, may poorly respond to ICIs [5, 6]. In contrast, NSCLC patients with *TP53*/*KRAS* co-mutations usually have remarkable clinical outcomes following treatment with anti-PD-1 or anti-PD-L1 immunotherapy. Hence, the clinical efficacy of ICI-based immunotherapy for NSCLC patients with oncogenic driver gene mutations is variable, suggesting that these oncogenic driver gene mutations may shape the immune landscape in the NSCLC microenvironment to modulate immune responses to NSCLC. Conceivably, evaluating how these oncogenic driver gene mutations modulate the immunotherapy response and the immune microenvironment in NSCLC patients is crucial.

The mutation in V-Raf murine sarcoma viral oncogene homolog B (BRAF) kinase has been implicated as an oncogenic driver in NSCLC. It can lead to cancer cell proliferation and survival. About 2–4% of patients with lung cancer carry *BRAF* mutations, predominantly in those with lung adenocarcinoma [7, 8]. Notably, treatment with a BRAF inhibitor, such as vemurafenib or dabrafenib, has achieved an objective response rate (ORR) of 33–42% and median progression-free survival (PFS) of 5.5 to 7.3 months in V600E *BRAF*-mutated NSCLC patients, respectively [9, 10]. Treatment with both dabrafenib and trametinib (MEK inhibitor) enhances the therapeutic efficacy and leads to an ORR of 64% and median PFS of 14.6 months in V600E *BRAF*-mutated NSCLC patients based on independent review committee assessment [11]. Although NSCLC patients with V600E *BRAF* mutations most likely benefit from BRAF inhibitors with MEK inhibitors, they eventually develop therapeutic resistance [12]. Furthermore, treatment with BRAF inhibitors is ineffective in most NSCLC patients with Non-V600E *BRAF* mutations. Thus, immunotherapies for *BRAF*-mutated NSCLC patients may be an attractive exploration. Based on the IMMUNOTARGET and GFPC 01-2018 studies, monotherapy with a PD-1 inhibitor has similar efficacy in both *BRAF*-mutated and wild-type NSCLC patients [13, 14]. In addition, previous studies have shown that *BRAF* mutations are associated with positive PD-L1 expression in NSCLC tissues [13–16]. Further analysis has reported that patients with *BRAF*-mutated lung cancer have low/intermediate tumor mutational burden (TMB) and microsatellite-stable status [16]. However, the effect of *BRAF* mutations on the immune microenvironment remains unclear.

Here, we performed Nanostring-panel RNA sequencing of 57 *BRAF*-mutated and wild-type tumor tissue specimens to explore the immune microenvironment. In addition, we retrospective studied the efficacy of monotherapy with an ICI and combined therapies with ICIs in 417 patients with wild type, and *BRAF*-mutated NSCLC in two centers.

## Materials and methods

### Patients and specimens

NSCLC patients were recruited at Zhejiang Cancer Hospital and Hunan Cancer Hospital from 01 June 2017 to 01 March 2022. The criteria for recruited NSCLC patients included: (1) Patients were pathologically confirmed NSCLC; (2) *BRAF* mutation status was determined by NGS using commercially available panels targeting 168 cancer-related genes and sequenced on a NextSeq 500 (Illumina, San Diego, CA) with paired-end reads with a target sequencing depth of 1000× for tissue samples using optimized protocols (Burning Rock Biotech, Guangzhou, China); (3) Patients had no sensitive *EGFR*, *ALK*, or *ROS1* driver gene alteration; (4) Patients in cohort B were diagnosed as locally advanced without radical radiotherapy or advanced NSCLC patients and received the ICIs treatment. And patients involved in cohort A were allowed in any stage; (5) Patients must have at least one measurable lesion diagnosed by computed tomography (CT) scans or brain magnetic resonance imaging (MRI) before the initiation of anti-PD-1/PD-L1 treatment in cohort B. Their baseline demographic and clinical characteristics, including PD-L1 expression and BRAF mutation status, were recorded. PD-L1 expression in naive treatment tumor biopsy samples was assessed using the Dako 22C3 platform (Agilent, Santa Clara, CA, USA). A patient was considered PD-L1 negative if <1% of tumor cells were stained positive. This study was approved by the Institutional Review Board of Zhejiang Cancer Hospital and Hunan Cancer Hospitals (ID: IRB-2020-240), and written informed consent was obtained from individual patients before enrollment. There were 57 formalin-fixed paraffin-embedded (FFPE) NSCLC tissue samples with either *BRAF* mutant or wild-type for Nanostring-panel RNA sequencing (cohort A). And the tumor biopsy samples of 57 patients in cohort A were all collected before any treatment. Another group of 417 NSCLC patients (cohort B) was recruited to test their responses to ICI monotherapy or combined therapies. Patients, who did not undergo the evaluation of therapeutic efficacy, or were lost to follow-up, were excluded.

### Assessments

The responses of individual patients to the therapies were evaluated by chest CT every two cycles of treatments, according to Response Evaluation Criteria in Solid Tumors version 1.1 (RECIST v1.1). Their therapeutic responses were defined as complete response (CR), partial response (PR), stable disease (SD), and progressive disease (PD). Those patients were regularly followed up. The primary endpoint was the overall survival (OS), defined as the time from the initiation of immunotherapy to death or the end of the last follow-up. The secondary endpoints included progression-free survival (PFS) and objective response rate (ORR). The PFS is the time between immunotherapy initiation and disease progression or death. The ORR referred to the proportion of patients with CR or PR, while the disease control rate DCR referred to patients with CR, PR, or SD.

### Nanostring-panel RNA sequencing

Biopsied tumor tissue specimens were obtained from the first cohort of patients before any specific treatment. RNA was isolated from dissected tumor tissue using an RNeasy FFPE kit (Qiagen, Valencia, CA). A tissue surface area of approximately 50 mm^2^ was used to harvest the necessary amount of RNA (~50 ng). RNA was input directly into the nCounter platform (NanoString Technologies, Seattle, WA) for the hybridization reaction [17]. Subsequent transcriptome analysis is based on the customized 289-immuno-gene panel (Supplemental Table 1), which includes 289 genes involved in the immune response in cancer for individual tumor samples. For each sample, quality control indicators, including the Imaging QC, Binding Density QC, Positive Control Linearity QC, Positive Control Limit of Detection QC, Positive normalization factor, and Content normalization factor, were then calculated. Samples that passed the quality control were included in the subsequent analysis. The NanoStringNorm package in R was used to normalize raw data. Especially, data was normalized against the geometric mean of five housekeeping genes in combination with a positive control normalization, which uses the geometric mean of six synthetic positive targets to control technical variability in the assay. The obtained gene expression values were then log2-transformed.

### Estimation of cell infiltration in the tumor immune microenvironment (TIME)

These tested gene profiles covered marker genes of 14 immune cell types, including B cells, dendritic cells (DCs), macrophages, exhausted CD8, T cells, CD8 T cells, neutrophils, mast cells, cytotoxic cells, Treg, CD56^dim^ NK cells, NK cells, CD45, and Th1 cells [18–20]. The macrophages were further divided into M1 and M2 macrophages [21, 22]. The cell infiltration scores in all TIME were calculated as the arithmetic means of the constituent genes [20].

### Generation of TIME signatures

For the study of TIME signatures, four signatures of (1) IFN-γ signature; (2) GEP score; (3) T cell markers; (4) Chemokines were achieved using a specific set of genes (Supplemental Table 2) with relevant biological function, respectively [23, 24]. The GEP score was calculated as a weighted linear average of the constituent genes while the remaining signatures were calculated as the arithmetic means of the corresponding gene [23].

### Measurements of pathway

To test the differences regarding the immune pathways, we extracted gene lists from the method previously reported [25]. For each gene list, we defined a score as the average gene expression based on log2 transformation. To determine the enrichment of expression of the three gene sets of the Molecular Signatures Database (MSigDB) Hallmark collection, we used the Gene Set Variation Analysis (GSVA) Bioconductor package [26, 27] to analysis.

### Statistical analysis

The data were statistically analyzed using SPSS 26.0 (IBM Corp., Armonk, New York, USA), GraphPad Prism 6.0 (GraphPad Software, San Diego, California, USA), and R software version 4.0.5 (R Foundation for Statistical Computing, https://www.R-project.org/). The difference in demographic, clinical, and pathologic characteristics between the two groups was analyzed by Fisher’s exact and Wilcoxon rank-sum tests. All two-group comparisons for scores use the two-sided Wilcoxon rank-sum test (R function Wilcox. test). Boxplots were generated in R with the ggplot2 package, indicating the median and interquartile range. Heatmaps were z-score scaled by row using the R package “Pheatmap.” Survival analyses were performed with Kaplan–Meier curves and log-rank test, and *p* value < 0.05 was used as a significant threshold in the remaining statistical analysis. To adjust for the possible selection bias induced by the retrospective, non-randomized design, differences in PFS and OS were also evaluated using inverse probability of treatment weighting (IPTW) analyses.

## Results

### *BRAF* mutation does not alter immune infiltrates in the NSCLC environment

**Fig. 1:**
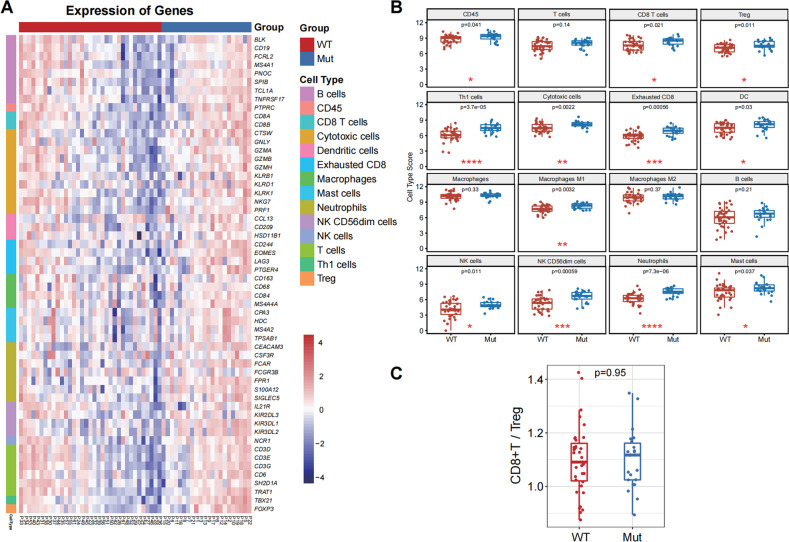
The immune cell scores in both wild-type and *BRAF*-mutated NSCLC. **A** Heatmap of gene transcripts related to various subsets of immune cells in the WT and *BRAF* mutated NSCLC specimens. **B** Quantitative analysis of immune cell scores in the WT and *BRAF* mutated NSCLC specimens. **C** The ratios of CD8 T cells to Tregs in the WT and *BRAF* mutated NSCLC specimens.

To explore whether *BRAF* mutation could modulate the immune infiltrate landscape in NSCLC tissues, NSCLC patients were recruited. After excluding any patients with co-mutations in *EGFR*, *ALK*, and *ROS1*, there were 22 NSCLC patients with only *BRAF* mutation and 35 NSCLC patients with wild-type *BRAF*, and their demographic and clinic characteristics are shown in Table 1. There were similar demographics and clinical feathers among these two groups. Analysis of biopsied tumor specimens revealed the gene sets related to T cells, CD8 T cells, exhausted CD8 T cells, Treg cells, B cells, macrophages, CD45, mast cells, DCs, NK cells, CD56^dim^ NK cells, cytotoxic cells, Th1 cells, and neutrophils between the BRAF wild-type (WT) and BRAF mutation (Mut) groups (Fig. 1A). Compared with the WT group, a higher level of immune infiltrate (CD45+ cells) related genes was detected in *BRAF*-mutated patients.

**Table 1:** Association of demographics and clinicopathological characteristics with BRAF mutation status in Cohort A.

Characteristics	Wild type (*n* = 35)	*BRAF* mutation (*n* = 22)	*P*
	*n* (%)	*n* (%)	
Age (yrs.)			0.40
≤65	23 (65.7)	12 (54.5)	
>65	12 (34.3)	10 (45.5)	
Gender			0.99
Female	8 (22.9)	5 (22.7)	
Male	27 (77.1)	17 (77.3)	
Smoking status			0.79
Never smoker	10 (28.6)	7 (31.8)	
Former/current smoker	25 (71.4)	15 (68.2)	
Stage^a^			0.71
I–III	8 (22.9)	6 (27.3)	
IV	27 (77.1)	16 (72.7)	
ECOG PS			0.20
0–1	35 (100.0)	21 (95.5)	
2	0 (0.0)	1 (4.5)	
Pathological type			0.07
Adenocarcinoma	35 (100.0)	20 (90.9)	
Other types	0 (0.0)	2 (9.1)	

^a^Using the 8th TNM staging classification.

Further analyses indicated no statistical significance in the levels of B cell, macrophage, M2 macrophage, and T cell-related gene transcripts between the WT and Mut groups. In contrast, the transcripts of genes related to effective immune cells (such as CD8 + T cells, NK cells, M1 macrophages, Cytotoxic cells, and Th1 cells) and immune suppressors (such as Tregs, mast cells, and neutrophils) were enriched in the Mut group (Fig. 1B). These results indicated a complicated function of *BRAF* mutation on immune cell subsets infiltrations. To comprehensively evaluate the immune cell infiltration dominantly involved in immunotherapy, we calculated the ratios of CD8 + T cell scores/Treg scores between the Mut and WT groups of patients (Fig. 1C). There was no significant difference in the proportions of CD8 + T cell scores/Treg scores between the Mut and WT groups of patients, suggesting a balance of immune effective and suppressive cells and that *BRAF* mutation may not affect overall immune infiltrates in the NSCLC environment.

**Fig. 2:**
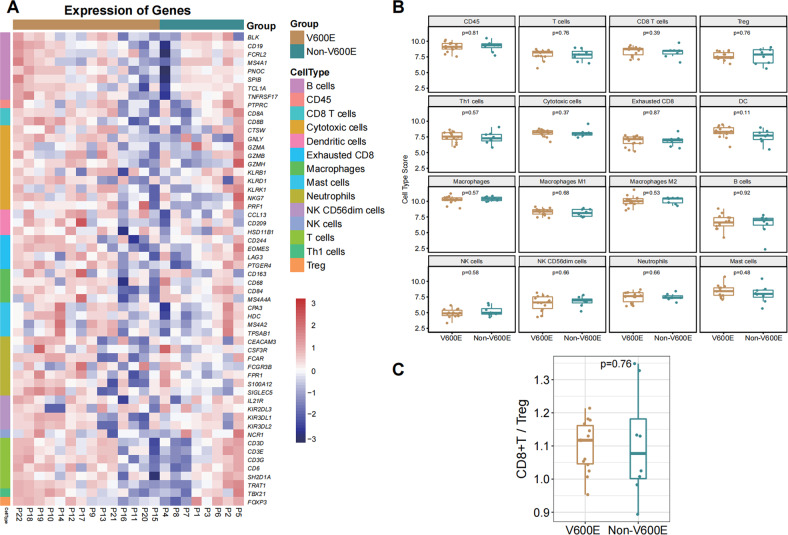
The immune cell scores in both *BRAF* V600E and Non-V600E NSCLC. **A** Heatmap of gene transcripts related to various subsets of immune cells in the *BRAF* V600E and Non-V600E NSCLC specimens. **B** Quantitative analysis of immune cell scores in the *BRAF* V600E and Non-V600E NSCLC specimens. **C** The ratios of CD8 T cells to Tregs in the *BRAF* V600E and Non-V600E NSCLC specimens.

We further determined the transcripts of immune infiltrate-related genes between 14 *BRAF* V600E and 8 Non-V600E NSCLC specimens (Fig. 2A). Their demographics and clinical feathers were similar (Supplemental Table 3). There was no significant difference in all types of cell scores between the V600E and Non-V600E NSCLC environment (Fig. 2B). Furthermore, there were also similar ratios of CD8 T cell scores to Treg scores between these two groups of specimens (Fig. 2C). Hence, the *BRAF* mutation did not affect immune cell infiltration in the NSCLC environment.

### *BRAF* mutation does not modulate the immune-related signal pathways in the NSCLC environment

**Fig. 3:**
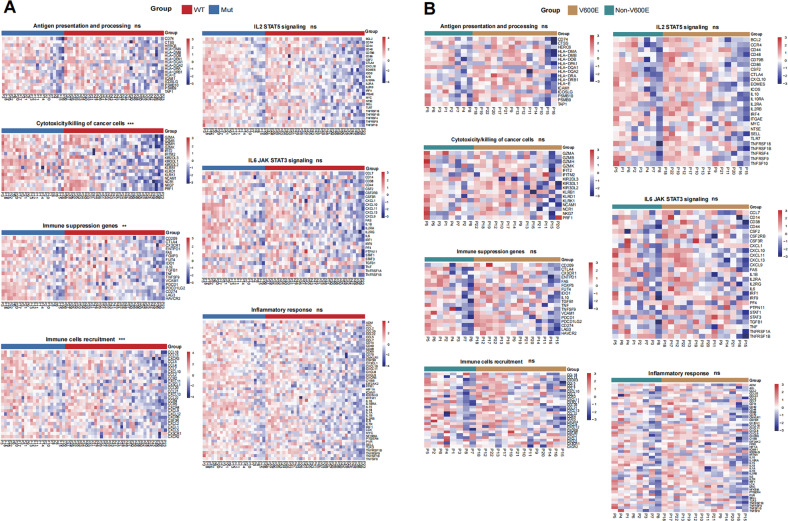
The effect of *BRAF* mutation on different immune-related signaling pathway in NSCLC. **A** Headmap analysis of gene transcripts involved in various immune pathways in the WT and *BRAF* mutated NSCLC specimens. **B** Heatmap analysis of genes transcripts involved in various immune pathways in the *BRAF* V600E and Non-V600E NSCLC specimens.

To better understand the potential role of *BRAF* mutation in anti-tumor immunity in the NSCLC microenvironment, we analyzed a series of genes related to the significant immune pathways. As shown in Fig. 3A, the transcripts of genes related to cancer cell cytotoxicity, immune suppression, and immune cell recruitment were upregulated in the *BRAF*-mutated NSCLC compared to wild-type patients. However, there was no significant difference in the transcripts of genes related to antigen processing and presentation, IL2-STAT5, IL6-JAK-STAT3 signaling, and inflammatory response between the WT and BRAF-mutated NSCLC tissues. Further analysis of the tumors with *BRAF* mutation revealed a similar level of gene transcripts between the *BRAF* V600E and Non-V600E NSCLC tumors (Fig. 3B).

### Immunotherapy-related scores and signatures between *BRAF*-mutated and WT NSCLC

**Fig. 4:**
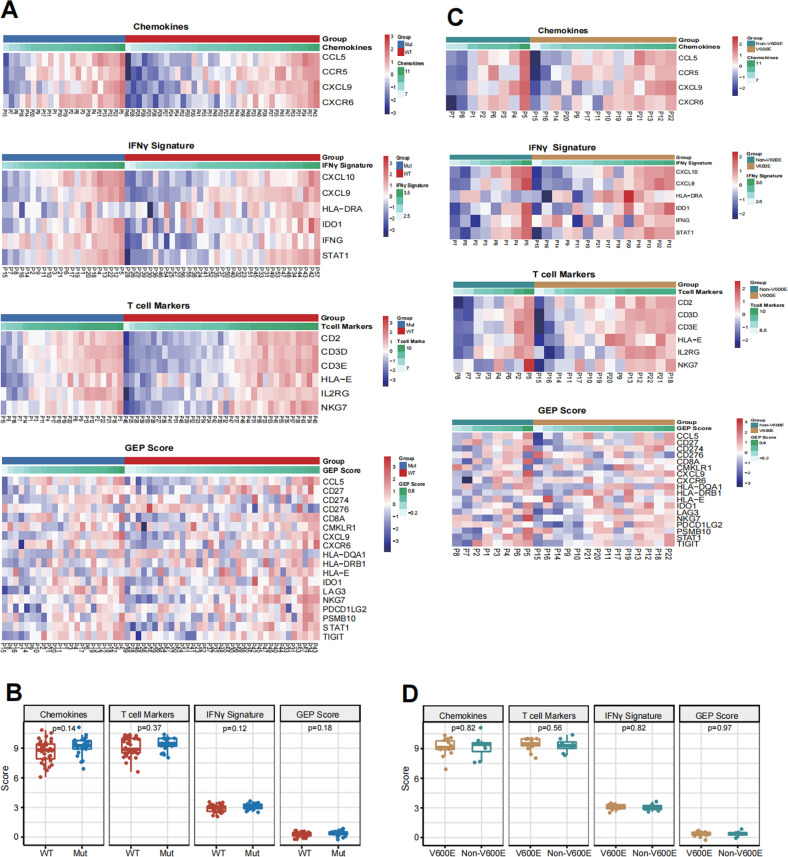
The effect of *BRAF* mutation on different immune signature in NSCLC. **A** Heatmap analysis of gene transcripts related to immune signatures in the WT and *BRAF* mutated NSCLC specimens. **B** Quantitative analysis of immune signatures in the WT and *BRAF* mutated NSCLC specimens. **C** Heatmap analysis of gene transcripts related to immune signatures in the *BRAF* V600E and Non-V600E NSCLC specimens. **D** Quantitative analysis of immune signatures in the *BRAF* V600E and Non-V600E NSCLC specimens.

According to the pathway analyses, we hypothesized the equal effect of *BRAF* mutation on the cytotoxic gene expression signatures and immune suppressive features. We used the GEP scores, based on 14 critical immune molecules (including positive and negative immune regulatory genes), to evaluate different immune signatures according to the gene expression profiling. The interferon γ (IFN-γ) signatures, T cell markers, and chemokines were also assessed by the gene expression profiling. There was no significant difference in the GEP score between *BRAF*-mutated and WT NSCLC groups, accompanied by similar IFN-γ signatures, T cell markers, and chemokines (Fig. 4A, B). Furtherly, there was no significant change in the transcripts of genes related to the immune signatures between the *BRAF* V600E and Non-V600E subgroups of NSCLC in this population (Fig. 4C, D). The data suggested that *BRAF* mutation did not alter the transcripts of genes involved in immunoregulation in the NSCLC environment. BRAF mutated, and WT NSCLC patients may have similar sensitivity to ICI treatment.

### The therapeutic responses of NSCLC patients to monotherapy and combined therapies with ICIs

**Fig. 5:**
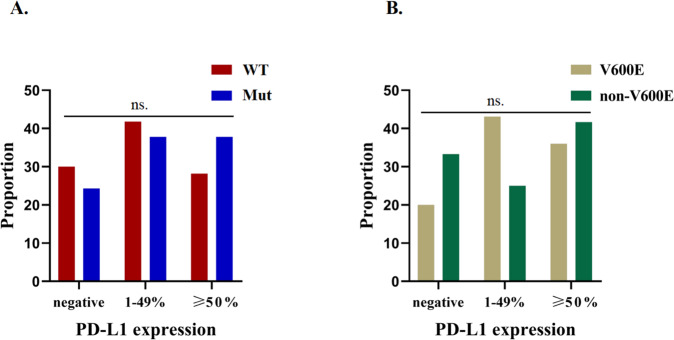
PD-L1 expression of patients according to BRAF mutation status and BRAF mutation type. **A** PD-L1 expression were evaluated in BRAF mutant and wild-type group. **B** PD-L1 expression were evaluated in BRAF V600E and Non-V600E subgroup.

In cohort B, 417 advanced NSCLC patients were treated with ICI monotherapy or combined therapies in Zhejiang Cancer hospital or Hunan Cancer hospital between June 2017 and March 2022. The cohort included 59 NSCLC patients with *BRAF* mutation (V600E, *n* = 43; Non-V600E, *n* = 16) and 358 NSCLC patients with WT *BRAF*. Their demographics and clinical feathers were similar, except for treatment lines (*p* = 0.01, Table 2). Following PD-L1 expression collected in 147 of all patients (147/417, 35.3%), there was no significant difference in the levels of PD-L1 expression between the WT (*n* = 110) and *BRAF* mutated groups (*n* = 37) (*P* = 0.92) (Fig. 5A and Table 2), as well as between the *BRAF* V600E (*n* = 25) and Non-V600E subgroups (*n* = 12) of patients (*P* = 0.49) (Fig. 5B and Supplemental Table 4). Moreover, a similar proportion of *BRAF* mutated and WT NSCLC patients received ICI monotherapy or combined therapies with ICIs (Table 2, *p* = 0.77).

**Table 2:** Association of demographics and clinicopathological characteristics with BRAF mutation status in Cohort B.

Characteristics	Wild type (*n* = 358)	*BRAF* mutation (*n* = 59)	*P*
	*n* (%)	*n* (%)	
Age (yrs.)			0.31
≤65	237 (66.2)	43 (72.9)	
>65	121 (33.8)	16 (27.1)	
Gender			0.36
Female	89 (24.9)	18 (30.5)	
Male	269 (75.1)	41 (69.5)	
Smoking status			0.33
Never smoker	128 (35.8)	25 (42.4)	
Former/current smoker	230 (64.2)	34 (57.6)	
Stage^a^			0.33
III	19 (5.3)	5 (8.5)	
IV	339 (94.7)	54 (91.5)	
ECOG PS			0.25
0–1	331 (92.5)	57 (96.6)	
2	27 (7.5)	2 (3.4)	
Pathological type			0.16
Adenocarcinoma	315 (88.0)	48 (81.4)	
Other types	43 (12.0)	11 (18.6)	
Treatment lines			0.01
First line	179 (50.0)	40 (67.8)	
Second/later line	179 (50.0)	19 (33.2)	
Treatment regimens			0.77
Monotherapy	116 (32.4)	18 (30.5)	
Chemotherapy plus ICIs	201 (56.1)	37 (62.7)	
Anti-angiogenesis plus ICIs	41 (11.2)	4 (6.8)	
PD-L1 expression			0.92^b^
Negative	33 (9.2)	9 (15.3)	
1–49%	46 (12.8)	14 (23.7)	
≥50%	31 (8.7)	14 (23.7)	
Unkonow	248 (69.3)	22 (37.3)	

aUsing the 8th TNM staging classification.

^b^Analysis in PD-L1 detected patients.

**Fig. 6:**
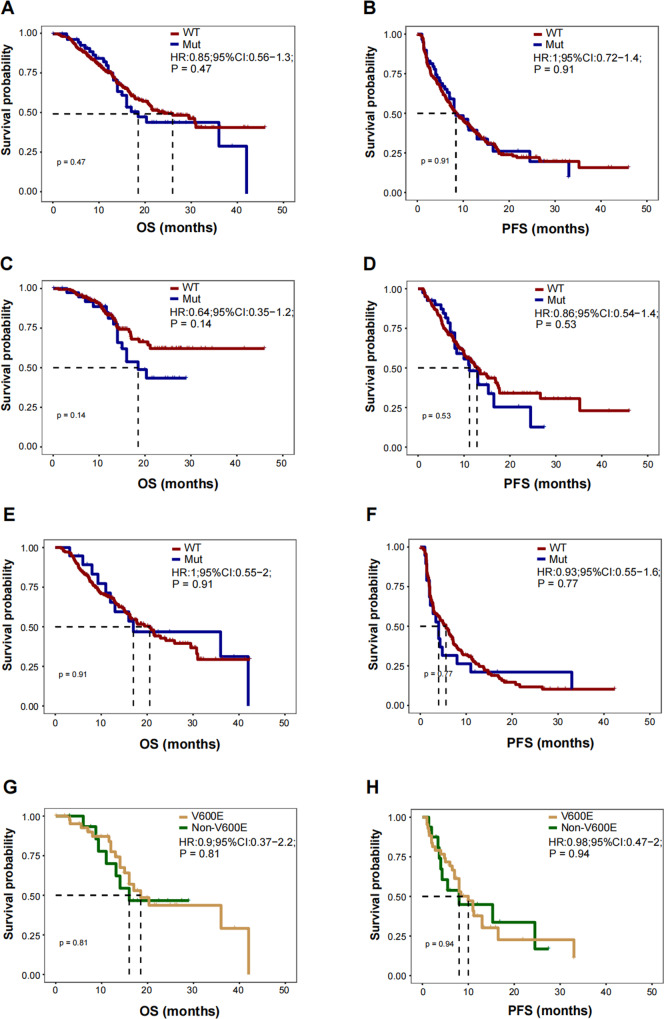
Kaplan–Meier curves for progression-free survival and overall survival with ICIs monotherapy or ICIs combined therapy. **A**, **B** Overall survival (**A**) and progression-free survival (**B**) with ICIs or ICIs combined therapy according to the BRAF mutation status. **C**, **D** Overall survival (**C**) and progression-free survival (**D**) for first line therapy according to the BRAF mutation status. **E**, **F** Overall survival (**E**) and progression-free survival (**F**) for second or later line therapy according to the BRAF mutation status. **G**, **H** Overall survival (**G**) and progression-free survival (**H**) with ICIs or ICIs combined therapy according to the BRAF mutation type.

After follow-up for a median of 16.2 months, 26 patients (44.1%) with *BRAF* mutated, and 125 patients (35.0%) with WT *BRAF* had died. The median OS for WT NSCLC patients was 18.5 months (95% CI: 13.7 to 23.2 months) and 26.0 months (95% CI: 19.4 to 32.5 months) for the BRAF mutated NSCLC patients. There was no significant difference in OS between WT and *BRAF*-mutated NSCLC patients following monotherapy or combined therapies with ICIs (HR: 0.85; 95% CI, 0.56 to 1.30; *p* = 0.47, Fig. 6A). Similarly, the median PFS for the WT BRAF NSCLC patients was 8.4 (7.0 to 9.9) months and 8.4 (5.3 to 11.5) months for the *BRAF* mutated NSCLC patients. As a result, there was no significant difference in PFS between the WT and *BRAF*-mutated NSCLC patients treated with ICIs monotherapy or combined therapy (HR: 1.00; 95% CI, 0.72 to 1.40; *p* = 0.91, Fig. 6B). During the study, 27 out of 59 *BRAF* mutated NSCLC patients experienced partial response or complete response with an ORR of 45.8%, and 118 out of 358 WT *BRAF* NSCLC patients had a partial response or complete response with an ORR of 33.0%, indicating a comparable response rate between these two groups of NSCLC patients (*p* = 0.056).

Next, we performed the subgroup analysis according to the therapy lines. For first-line therapy (*n* = 218), there was no significant difference in OS (NA vs. 18.5 m, HR: 0.64; 95% CI, 0.35 to 1.20; *p* = 0.15), PFS (12.8 m vs. 11.2 m, HR: 0.86; 95% CI, 0.54 to 1.40; *p* = 0.53) and ORR (45.3% vs. 57.5%, *p* = 0.16) (Fig. 6C, D) between WT NSCLC and the *BRAF* mutated patients. Similar OS (20.6 m vs. 17.0 m, HR: 1.00; 95% CI, 0.55 to 2.00; *p* = 0.91), PFS (5.6 m vs. 4.0 m, HR: 0.93; 95% CI, 0.55 to 1.60; *p* = 0.77) and ORR (20.7% vs. 21.1%; *p* = 0.97) were also observed between these two groups in second or later line therapy (Fig. 6E, F). Importantly, further stratification analysis of patients with *BRAF* mutations, according to *BRAF* mutation type, revealed that similar OS (18.5 vs. 16.0, HR: 0.90; 95% CI, 0.37 to 2.20; *p* = 0.81), PFS (10.0 vs. 8.0, HR: 0.98; 95% CI, 0.47 to 2.00; *p* = 0.94) and ORR (51.7% vs. 31.1%; *p* = 0.17) between the *BRAF* V600E and Non-V600E NSCLC patients (Fig. 6G, H, Supplemental Table 4).

On the other hand, we also use the inverse probability of treatment weighting (IPTW) analyses to evaluate the PFS and OS in cohort B to adjust for the possible selection bias induced by the retrospective, non-randomized design. There was no significant difference in PFS and OS between WT and *BRAF*-mutated NSCLC patients (Supplemental Fig. 1A, B). Consistently, the subgroup analysis according to therapy lines showed similar PFS and OS in two groups (Supplemental Fig. 1C, D, first-line therapy; Supplemental Fig. 1E, F, second or later-line therapy). Meanwhile, the PFS and OS between the *BRAF* V600E and Non-V600E NSCLC patients were similar (Supplemental Fig. 1G, H).

## Discussion

Immunotherapy for advanced NSCLC patients with oncogenic driver gene mutations remains a challenge, as whether oncogenic driver gene mutation affects the TIME in NSCLC has not been clarified. Here, we present our immunogenomic and clinical study data in a patient population. Despite a higher level of immune cell infiltration, *BRAF*-mutated tumors had similar ratios of CD8 + T cells to Tregs and a balance of cytotoxicity gene expression signatures and immune suppressive features. Furthermore, patients with *BRAF*-mutated NSCLC had equivalent ICI-response-related biomarkers, such as the GEP scores and IFN-γ signature, to those with WT *BRAF* NSCLC. Moreover, similar immunophenotypes were detected both in *BRAF* V600E and Non-V600E NSCLC. Consistent with these findings, the further study confirmed that NSCLC patients with *BRAF* mutations displayed similar sensitivity to ICI-based monotherapy or combined therapies compared with those with WT *BRAF*. To the best of our knowledge, our work is the first report on the immunophenotype in *BRAF* mutated NSCLC. Our findings support that *BFAF*-mutated and WT NSCLC patients respond similarly to ICIs.

Previous retrospective studies with small sample sizes have explored some molecular characteristics that predict the efficacy of immunotherapy for *BRAF*-mutated NSCLC patients [16, 28]. An analysis of 29 patients reported that *BRAF* mutation was related to low/intermediate TMB and microsatellite-stable status [16]. In addition, 69% (20/29) and 40% (13/29) of patients were detected with PD-L1 positive expression (PD-L1 ≥ 1%), and high PD-L1 expression (PD-L1 ≥ 50%) in these 29 NSCLC patients harbored BRAF mutation [16]. Despite PD-L1 expression, TMB, and microsatellite-stable status, the TIME in *BRAF*-mutated NSCLC, which is crucial for patients responding to ICIs, has never been evaluated. We performed Nanostring-panel RNA sequencing in this study to explore the TIME in *BRAF*-mutated and WT NSCLC specimens. Interestingly, we found that the transcripts of genes related to anti-tumor immune cells such as CD8 + T cells, and immunosuppressive cell such as Tregs were simultaneously enriched in *BRAF*-mutated NSCLC, suggesting that *BRAF* mutation may play a complicated role in the infiltration of different subsets of immune cells. Further analysis revealed a similar ratio of CD8 + T cells score to Treg score between the *BRAF*-mutated and WT NSCLC patients, indicating balanced recruitment between immune effector cells and suppressive cells driven by *BRAF* mutation. Moreover, the transcripts of genes related to cytotoxicity/killing of cancer cells and immune suppression, but no antigen processing and presentation, the JAK-STAT and IFN-γ signaling were enriched in *BRAF*-mutated NSCLC. Accordingly, we speculate that *BRAF* mutation may similarly modulate the cytotoxic gene expression signatures and immune suppressive features in NSCLC. Meanwhile, combined with the analysis of immune-related scores (GEP scores, IFN-γsignatures, T cell markers, and chemokines) in our results, we could infer that *BRAF* mutation may result in balanced immunomodulatory effects and did not affect the therapeutic responses to ICIs in NSCLC patients.

Several single-arm, retrospective studies reported 69–76% of *BRAF*-mutant patients were found with positive PD-L1 expression(PD-L1 ≥ 1%), and 38–57% of patients were detected with high PD-L1 expression (PD-L1 ≥ 50%) [13–16]. Owing to lacking *BRAF* wild-type population group, these research did not run the statistical analysis according to the *BRAF* mutation status. Further, Chenxing Zhang et al. showed that similar PD-L1 expressions were detected in *BRAF* mutant and wild-type NSCLC patients (*p* = 0.198) [28]. Our results presented that 75.7% and 70% of patients were PD-L1 positive separately in *BRAF*-mutant and wild-type groups, and there was no difference in each group, which was consistent with the above report. However, the distribution of PD-L1 expression in the *BRAF* V600E subgroup and *BRAF* Non-V600E were controversial. Dudnik et al. found that a higher proportion of positive PD-L1 expression was detected in the V600E (14/19, 74%) subgroup compared to Non-V600E (6/10, 60%) (*P* = 0.05), but a similar proportion in high PD-L1 expression status (42% vs. 50%) [16]. The GFPC 01-2018 study showed that 71% of BRAF V600E patients had high PD-L1 expression, compared with 29% in the *BRAF* Non-V600E subgroup [13]. However, another study reported *BRAF* Non-V600E subgroup (3/5, 60%) exhibited higher proportion of high PD-L1 expression compared to *BRAF* V600E subgroup (2/8, 25%) [15]. These studies all had small sample sizes, and the effects of different mutation types of BRAF on PD-L1 expression were not consistent. In our study, there was no difference in the distribution of PD-L1 expression between the *BRAF* V600E and Non-V600E subgroups. This observation was consistent with the clinical outcomes of each subgroup treated with ICIs. And the effect of *BRAF* mutation on PD-L1 expression still needs more studies, including meta-analysis.

The Memorial Sloan Kettering-Integrated Mutation Profiling of Actionable Cancer Targets (MSK-IMPACT) study revealed that treatment with ICIs resulted in a similar OS in 27 *BRAF* mutated and 323 WT NSCLC patients with a median OS 10 m vs. 11 m, *P* = 0.334 [28]. A retrospective study from an Italian expanded-access program enrolled 1588 advanced non-squamous NSCLC patients treated with second-line Nivolumab. A similar OS was observed in *BRAF*-mutant (*n* = 11), *BRAF* wild type (*n* = 199), and *BRAF* not evaluated group (*n* = 1378), which were 10.3, 11.2, and 11.0 months, respectively [29]. Our findings were consistent with these observations that *BRAF*-mutated NSCLC patients have similar responses to ICI monotherapy compared to WT *BRAF*. Further, our results also unveiled that ICIs combined with chemotherapy or anti-angiogenesis regimens had similar objective response rates or survival regardless of *BRAF* mutations. Based on our TIME analysis and clinical investigation, we proposed that patients with NSCLC harboring *BRAF* mutation have comparable clinical benefits from ICIs therapy compared to *BRAF* wild-type patients.

Interestingly, the sensitivity of *BRAF* V600E and Non-V600E NSCLC to ICIs was controversial. Elizabeth Dudnik et al. found that *BRAF* mutation in NSCLC did not affect the objective response rate (25% vs. 33%, *P* = 1.0) and PFS (3.7 m vs. 4.1 m, *P* = 0.37) [16]. Conversely, another study reported a superior OS in *BRAF* Non-V600E subgroup compared to the *BRAF* V600E subgroup of NSCLC patients (5.0 vs. 14.0 m, *P* = 0.017) [28]. The small sample size may cause these controversial results in both studies, including 22 and 27 *BRAF*-mutated NSCLC patients separately. Our TIME analysis exhibited similar immune cell infiltrations, immune-related gene transcripts, and ICIs response predictive scores and signatures, supporting a comparable clinical benefit from ICIs between the *BRAF* V600E and Non-V600E subgroups of NSCLC patients. Further validation in the cohort B analysis also revealed similar ORR, PFS, and OS in both *BRAF* V600E and Non-V600E subgroups of NSCLC patients following treatment with ICIs monotherapy and combined with ICIs. Our findings suggest that NSCLC patients may respond to immunotherapy regardless of *BRAF* mutation type. Thus, although NSCLC patients with Non-V600E *BRAF* mutation are resistant to BRAF inhibitors, they still respond to ICI-based immunotherapy.

For patients with *BRAF* V600E mutation, the optimal therapy is still unclear. Undoubtedly, NSCLC patients benefit more significantly from first-line immunotherapy than second or later-line immunotherapy [4]. However, there seems to be little difference in clinical benefits from BRAF-targeted therapy or BRAF plus MEK inhibitors in the first line and second or later line setting. Given that combination of ICIs and chemotherapy resulted in an ORR of 63.3%, median PFS of 11.0 months, and median OS of 20.3 months in *BRAF* V600E mutated patients in our study, we propose that the optimal sequence of the therapy regimen may be chemotherapy plus ICIs as the first-line therapy and then targeted therapy for *BRAF* V600E NSCLC patients. However, whether treatment with a BRAF inhibitor or BRAF plus MEK inhibitors would shift the balance of immune effective and suppressive factors associated with *BRAF* mutation and whether post-targeted tumors would be more or less likely to respond to ICB remain to be investigated. Previous studies revealed that immunotherapy followed by EGFR-TKI therapy caused a higher incidence of immune-related pneumonia than EGFR TKI followed ICIs [30, 31]. The safety of different sequences of therapy regimens should also be evaluated. A stage Ib study including 28 NSCLC patients regardless of *BRAF* status explored the efficacy and safety of ICIs plus MEK inhibitor and reported that the median OS was 13.2 m, and the ORR was 18% [32]. Interestingly, ICI plus *BRAF-*targeted therapy is promising in melanoma patients [33], but the safety of this combined regimen is warranted. In addition, several clinical trials aimed to explore the efficacy and safety of targeted therapy plus ICIs in NSCLC patients are going [34].

Our study had several limitations. First, the fewer biopsied specimens limited our ability to detect the extent of immune feathers related to ICIs. The small sample size limited the degree of evidence of our tumor microenvironmental results, and research with bigger sample size are needed to confirm our results. Second, TMB was not evaluated in our study due to the small amount of tissue in biopsy specimens. Third, Nanostring-RNA sequencing was only performed in 20% of patients following treatment with ICIs; thus, TIME analysis and immunotherapy efficacy have not been investigated in the same patients. Again, our panel included only 289 immune-related genes; therefore, further specific and detailed analysis of the effect of *BRAF* mutation on TIME still needs additional translational and preclinical experiments.

## Conclusion

In summary, we found that *BRAF* mutation, including V600E, caused a balanced immunomodulatory effect with similar TIME compared to wild type NSCLC. Consistent with TIME analysis, our retrospective study confirmed that NSCLC patients had similar responses to ICIs monotherapy or combined therapy with ICIs regardless of *BRAF* mutation status. Our work supports that patients with NSCLC harboring *BRAF* mutation should not be denied treatment with ICIs.

## Supplementary information

Supplemental Figure 1

Supplemental Table 1

Supplemental Table 2

Supplemental Table 3

Supplemental Table 4

Supplemental Figure Legends

Reproducibility Checklist

## Data Availability

The Nanostring-panel RNA sequencing and other data have been deposited into OMIX database (https://ngdc.cncb.ac.cn/omix/) with Accession ID: OMIX001548 for Cohort A and OMIX001549 for Cohort B. All other data supporting the findings of this study are available from the corresponding author on reasonable request.
