# Supplementary figures and images for: Tumor immune microenvironment and immunotherapy efficacy in BRAF mutation non-small-cell lung cancer
Source: Cell Death Dis. 2022 Dec 21;13(12):1064. doi: 10.1038/s41419-022-05510-4 (PMC9772302; doi:10.1038/s41419-022-05510-4)

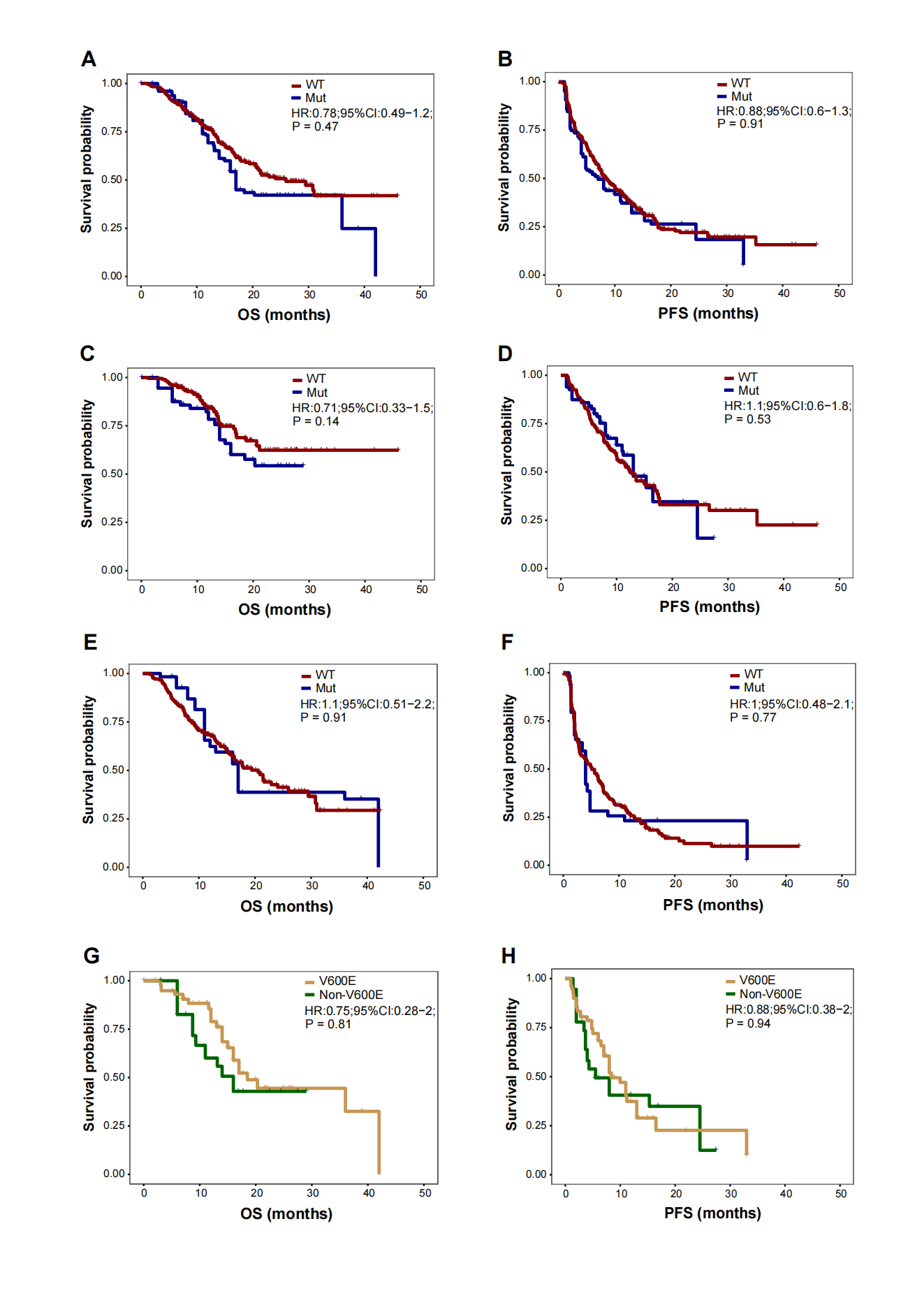

Supplement: Supplementary file 1 — Supplemental Figure 1 [file 41419_2022_5510_MOESM1_ESM.tif]
