# Supplementary material for: Tumor immune microenvironment and immunotherapy efficacy in BRAF mutation non-small-cell lung cancer
Source: Cell Death Dis. 2022 Dec 21;13(12):1064. doi: 10.1038/s41419-022-05510-4 (PMC9772302; doi:10.1038/s41419-022-05510-4)
Supplement: Supplementary file 3 — Supplemental Table 2 [file 41419_2022_5510_MOESM3_ESM.docx]

| **TIME signatures** | **Selected genes** |
| --- | --- |
| **IFN-γ signature** | *CXCL10, CXCL9, HLA-DRA, IDO1, IFNG, STAT1* |
| **GEP score** | *TIGIT, CD27, CD8A, PDCD1LG2, LAG3, CD274, CXCR6, CMKLR1, NKG7, CCL5, PSMB10, IDO1, CXCL9, HLA-DQA1, CD276, STAT1, HLA-DRB1, HLA-E* |
| **T cell markers** | *CD2, CD3D, CD3E, HLA-E, IL2RG, NKG7* |
| **Chemokines** | *CCL5, CCR5, CXCL9, CXCR6* |

**Supplemental Table2. The genes used in TIME signatures analysis.**
