# Supplementary material for: Tumor immune microenvironment and immunotherapy efficacy in BRAF mutation non-small-cell lung cancer
Source: Cell Death Dis. 2022 Dec 21;13(12):1064. doi: 10.1038/s41419-022-05510-4 (PMC9772302; doi:10.1038/s41419-022-05510-4)
Supplement: Supplementary file 4 — Supplemental Table 3 [file 41419_2022_5510_MOESM4_ESM.docx]

| **Supplemental Table3. Association of demographics and clinicopathological characteristics with *BRAF* mutation type in Cohort A.** | | | |
| --- | --- | --- | --- |
| **Characteristics** | ***BRAF* V600E**  **(n=14)** | ***BRAF***  **Non-V600E**  **(n=8)** | ***P*** |
|  | **n (%)** | **n (%)** |  |
| **Age (yrs.)** |  |  | 0.67 |
| **≤ 65** | 7 (50.0) | 5 (62.5) |  |
| **> 65** | 7 (50.0) | 3 (37.5) |  |
| **Gender** |  |  | 0.35 |
| **Female** | 5 (35.7) | 1 (12.5) |  |
| **Male** | 9 (64.3) | 7 (87.5) |  |
| **Smoking status** |  |  | 0.19 |
| **Never smoker** | 6 (42.9) | 1 (12.5) |  |
| **Former/current smoker** | 8 (57.1) | 7 (87.5) |  |
| **Stage*** |  |  | 0.35 |
| **II-III** | 5 (35.7) | 1 (12.5) |  |
| **IV** | 9 (64.3) | 7 (87.5) |  |
| **ECOG PS** |  |  | 1 |
| **0-1** | 13 (92.9) | 8 (100.0) |  |
| **2** | 1 (7.1) | 0 (0.0) |  |
| **Pathological type** |  |  | 1 |
| **Adenocarcinoma** | 13 (92.9) | 7 (87.5) |  |
| **Other types** | 1 (7.1) | 1 (12.5) |  |

*Using the 8^th^ TNM staging classification.
