# Supplementary material for: Tumor immune microenvironment and immunotherapy efficacy in BRAF mutation non-small-cell lung cancer
Source: Cell Death Dis. 2022 Dec 21;13(12):1064. doi: 10.1038/s41419-022-05510-4 (PMC9772302; doi:10.1038/s41419-022-05510-4)
Supplement: Supplementary file 5 — Supplemental Table 4 [file 41419_2022_5510_MOESM5_ESM.docx]

| **Supplemental Table4. Association of demographics and clinicopathological characteristics with *BRAF* mutation type in Cohort B** | | | |
| --- | --- | --- | --- |
| **Characteristics** | ***BRAF***  **V600E**  **(n=43)** | ***BRAF* Non-V600E**  **(n=16)** | ***P*** |
|  | **n (%)** | **n (%)** |  |
| **Age (yrs.)** |  |  | 0.08 |
| **≤65** | 34 (79.1) | 9 (56.3) |  |
| **> 65** | 9 (20.9) | 7 (43.8) |  |
| **Gender** |  |  | 0.07 |
| **Female** | 16 (37.2) | 2 (12.5) |  |
| **Male** | 27 (62.8) | 14 (87.5) |  |
| **Smoking status** |  |  | 0.64 |
| **Never smoker** | 19 (44.2) | 6 (37.5) |  |
| **Former/current smoker** | 24 (55.8) | 10 (62.5) |  |
| **Stage*** |  |  | 0.15 |
| **III** | 5 (11.6) | 0 (0.0) |  |
| **IV** | 38 (88.4) | 16 (100.0) |  |
| **ECOG PS** |  |  | 0.38 |
| **0-1** | 41 (95.3) | 16 (100.0) |  |
| **2** | 2 (4.7) | 0 (0.0) |  |
| **Pathological type** |  |  | 0.13 |
| **Adenocarcinoma** | 37 (86.0) | 11 (68.8) |  |
| **Other types** | 43 (14.0) | 11 (31.3) |  |
| **Treatment lines** |  |  | 0.60 |
| **First line** | 30 (69.8) | 10 (62.5) |  |
| **Second/later line** | 13 (30.2) | 6 (37.5) |  |
| **Treatment regimens** |  |  | 0.15 |
| **Monotherapy** | 11 (25.6) | 6(37.5) |  |
| **Chemotherapy plus ICIs** | 31 (72.1) | 8 (50.0) |  |
| **Anti-angiogenesis plus ICIs** | 1 (2.3) | 2 (12.5) |  |
| **PD-L1 expression** |  |  | 0.49^&^ |
| **negative** | 5 (11.6) | 4 (25.0) |  |
| **1-49%** | 11 (25.6) | 3 (18.8) |  |
| **≥50%** | 9 (20.9) | 5 (31.3) |  |
| **Unkonown** | 18 (41.9) | 4(25.0) |  |

*Using the 8^th^ TNM staging classification.

^&^ Analysis in PD-L1 detected patients
