# Supplementary material for: Tumor immune microenvironment and immunotherapy efficacy in BRAF mutation non-small-cell lung cancer
Source: Cell Death Dis. 2022 Dec 21;13(12):1064. doi: 10.1038/s41419-022-05510-4 (PMC9772302; doi:10.1038/s41419-022-05510-4)
Supplement: Supplementary file 6 — Supplemental Figure Legends [file 41419_2022_5510_MOESM6_ESM.docx]

**Supplemental Figure1** Inverse probability of treatment weighting (IPTW) analyses for progression-free survival and overall survival with ICIs monotherapy or ICIs combined therapy.

**A-B** Overall survival (**A**) and Progression-free survival (**B**) with ICIs or ICIs combined therapy according to the *BRAF* mutation status.

**C-D** Overall survival (**C**) and Progression-free survival (**D**) for first line therapy according to the *BRAF* mutation status.

**E-F** Overall survival (**E**) and Progression-free survival (**F**) for second or later line therapy according to the *BRAF* mutation status.

**G-H** Overall survival (**G**) and Progression-free survival (**H**) with ICIs or ICIs combined therapy according to the *BRAF* mutation type.
